# Ideas for mentorship in oncology for medical students and early career doctors: insights from a UK-wide oncology mentorship scheme

**DOI:** 10.1186/s12909-025-07219-2

**Published:** 2025-07-21

**Authors:** Joanna Kucharczak, Emma G. Khoury, Yarden Toiber Kent, Rahul Winayak, Simon Duke

**Affiliations:** 1 Department of Oncology, University of Cambridge, Cambridge, CB2 0XZ UK; 2 Academic Cancer Sciences Unit, University Hospital Southampton, Southampton, SO16 6YD UK; 3 Imperial College School of Medicine, Imperial College London, London, SW7 5 NH UK; 4 Kent Oncology Centre, Maidstone and Tunbridge Wells NHS Trust, Maidstone, ME16 9QQ UK

**Keywords:** Mentoring, Education, Oncology, Medical student, Undergraduate, Early career, Research

## Abstract

**Background:**

Early mentorship in oncology increases interest in the field and benefits mentees and mentors both personally and professionally. The British Oncology Network for Undergraduate Societies (BONUS) Mentorship Scheme enables UK-wide pairing of students and early career doctors with trainee and consultant oncologists. Little is known about the content of the sessions. Here we describe the objectives, contents, and outcomes of the scheme. We provide ideas and aims that mentees and mentors can utilize for future mentoring.

**Methods:**

A total of 101 mentors and 150 mentees were recruited from October 2022 to April 2023. Data were collected via pre-, mid-, and post-mentorship questionnaires. The questionnaires assessed the motivations, benefits, and improvements for the scheme with an overlying focus on the contents of the sessions. Qualitative data were analyzed via the framework method.

**Results:**

The main expectations for mentees were to become involved in research and receive oncology career guidance. The mentors’ main motivation was to promote oncology as a career. The most desirable traits for a mentor reported by mentees were being approachable and proactive.

The contents of the sessions consisted of discussions of the oncology career pathway, clinical shadowing, research projects and audits, discussions of oncology from clinical perspectives, signposting to career building opportunities, formal teaching, journal clubs and case discussions.

Mentees enjoyed the scheme and found participation valuable. As a result, they became more interested in oncology. The benefits described by mentees included exposure to oncology and confirmation of oncology as the right career choice. Personal satisfaction was the most frequently reported benefit for mentors. Interestingly, we found a mismatch between mentees’ desire for research opportunities and the scheme’s ability to facilitate this.

**Conclusions:**

Our study provides a comprehensive guide for mentorship ideas in undergraduate oncology. Our findings confirm the success of the BONUS Mentorship Scheme. We identify areas for future work, such as addressing the gap in cancer research opportunities for students and pre-specialty doctors.

**Supplementary Information:**

The online version contains supplementary material available at 10.1186/s12909-025-07219-2.

## Background

Training within oncology is in crisis with a shortfall of oncologists [1]. It is imperative that undergraduate medical students and early career doctors have opportunities to gain experience of the specialty. The positive impact of mentorship for both trainees and senior clinicians across medicine has been highlighted, notably leading to increased research productivity and insight into specific careers [2–4]. North American mentorship schemes in oncology for medical students and resident doctors are successful in recruiting to the specialty, promoting career development and increasing professional satisfaction [5–7]. Although several studies have focused on the structures and evaluations of mentorship schemes, very few focus on the description of contents, including activities and discussions, that take place during mentoring [8, 9].

The British Oncology Network for Undergraduate Societies (BONUS) implemented a United Kingdom (UK)-wide mentorship scheme in 2021. The objectives are to provide an overview of oncology and increase interest in and exposure to the specialty for undergraduate students and pre-specialty doctors. The scheme was set up due to an identified lack of exposure to oncology at an undergraduate level, which in the UK, consists of an average placement duration of 1–2 weeks and the mean quantity of clinic and ward-based teaching of 7.5 h [10, 11]. The BONUS Mentorship Scheme pairs medical students and resident doctors up to core training year 2, with specialty registrars and consultants in clinical, medical, surgical or interventional oncology. The mentorship takes place over a period of six months. Previous analyses of the BONUS Mentorship Scheme and another pilot scheme highlighted that they increase mentees’ knowledge of oncology and promote interest in the field [12, 13]. Similar schemes have been established in the UK in other specialties, including psychiatry and surgery [14–16].

As outlined by Fulton-Ward et al., their evaluation of the 2021–2022 cycle of the BONUS Mentorship Scheme did not investigate the specific content areas discussed within mentoring sessions [13]. They recommended that future work could use qualitative methodologies to investigate specific areas of content that mentors and mentees benefit from. To this end, we used web-based surveys to comprehensively study the contents of the 2022–2023 cycle of the BONUS Mentorship Scheme. We specifically sought to investigate the type and range of activities that took place during the mentoring sessions. We evaluated the feasibility of and presented content ideas for those wanting to engage with undergraduate oncology mentoring. We outlined how the scheme informs current health professional mentoring models [17]. Survey methods allowed us to collect both quantitative and qualitative data. Through surveys, we asked participants about their individual experience, which directly informed the aims of our study. A thorough analysis of the activities undertaken during mentoring sessions adds new knowledge and provides ideas for those wishing to undertake formal or informal mentoring within oncology.

## Methods

### Structure of the scheme

Recruitment to the mentorship scheme began in August 2022. Mentors were recruited across medical, clinical, surgical, and interventional oncology specialties. Eligible mentors were of Consultant or Specialty Registrar grade (i.e. a doctor in specialty training), or equivalent. Advertisement to mentors and mentees took place via email, social media platforms, and word of mouth. A link to a registration form was provided. The application period for mentees commenced in September 2022. Undergraduate medical students and pre-specialty resident doctors submitted a written application outlining why they would like to participate in mentoring. The pairing of mentors and mentees was based on the preferred location and preferred sub-specialty. The mentee was provided with contact details of the mentor, and it was their responsibility to contact their allocated mentor to arrange suitable times to meet. The official mentorship period took place from 1 October 2022 to 31 March 2023.

Initially, 150 mentees were paired with 101 mentors. During the scheme, 5 mentors and 3 mentees withdrew their participation. After the scheme finished, 80 mentors and 97 mentees confirmed full participation throughout the scheme. Mentors generally agreed to take on up to two or three mentees each. The participants were based in 24 locations across the UK and in a small number of overseas areas.

Mentees were encouraged to meet with their mentor one-on-one at least three times during the six-month duration of the scheme. The sessions were carried out online and in person and were individually agreed upon by the mentor and the mentee. Basic written guidance was provided via email to the participants prior to starting, which consisted of the following ideas: insights into a career in oncology, shadowing in a clinical setting, discussion of case studies and research papers, general career advice, engagement in research projects, networking opportunities, and teaching on oncology topics. The participants were additionally signposted to mentoring resources provided by the Academy of Medical Sciences [18].

### Data collection and analysis

All 150 mentees were invited to complete the following online web-based surveys: a pre-mentorship questionnaire in September 2022, a mid-mentorship questionnaire in November 2022, a mid-mentorship questionnaire in February 2023 and a post-mentorship questionnaire in April 2023. All 101 mentors were invited to fill out the pre-mentorship questionnaire in September 2022 and post-mentorship questionnaire in April 2023. The participants were sent regular email reminders to complete the questionnaires.

The questionnaires were composed of a mix of multiple choice and free text questions and aimed to assess the participants’ demographics, as well as the expectations, aims, concerns, contents and benefits of the scheme. The survey items were designed by the lead author. Inspiration was sought from surveys from previous similar studies [10, 13]. The survey was pretested by the senior author, who has expertise in medical education research. No previously validated survey instruments were used in this study. The final survey instruments can be found in Additional file 1.1 to 1.5.

The primary aim of the questionnaires was to collect data on the contents of the individual mentorship sessions. All the questionnaires were anonymous. Each question was optional, and no compensation or other incentives were provided to take part. Informed consent was obtained to process the data for research purposes. Regulatory approval from the Research Ethics Committee was waived.

Data collection closed in May 2023 and data analysis took place thereafter. Descriptive statistics, in the form of frequency, were used in the analysis of categorical data. Chi-square tests were applied to contingency tables where the samples were larger than 5. Fisher’s exact tests were applied where the samples were less than 5. Analyses were carried out in R 4.2.1 statistical software.

Framework analysis was undertaken for the qualitative free text data. Four of the authors familiarized themselves with the data by reading all the entries. Participant responses were then randomly allocated to two reviewers each, who then freely chose fitting themes to each response, without any predetermined constraints. The lead author then examined and compared the two themes and chose the best common fitting theme, given that the two themes were similar. Any discrepancies between the two chosen themes were discussed and resolved by all the contributors. In this study, we additionally report the total number and frequencies of themes that were identified per each free text question.

## Results

### Baseline characteristics

The survey response rates and career stages of the participants are summarized in Tables 1 and 2. Prior to the scheme, the most common experience in oncology among mentees was via lectures and formal teaching (*n* = 68, 91.9%), prior involvement in a research project (*n* = 21, 28.4%), oncology placement at medical school (n = 17, 23.0%), other non-oncology placements at medical school (*n* = 17, 23.0%), volunteering (*n* = 12, 16.2%) and participation in the previous cycle of the BONUS Mentorship Scheme (*n* = 11, 14.9%). There were no statistically significant differences in these parameters between pre-clinical students, clinical students and junior doctors, except for oncology and non-oncology placements, which were more significant in the clinical and doctor groups (Additional file 1.6).

**Table 1:** Response rates and career stages of mentees

	Pre-questionnaire: September 2022 (*n* = 74)	Mid-questionnaire: November 2022 (*n* = 27)	Mid-questionnaire: February 2023 (*n* = 23)	Post-questionnaire: April 2023 (*n* = 34)
Response rate	74/150 (49.3%)	27/150 (18.0%)	23/150 (15.3%)	34/150 (22.7%)
Pre-clinical student	31 (41.9%)	14 (51.9%)	10 (43.5%)	10 (29.4%)
Clinical student	40 (54.1%)	13 (48.1%)	12 (52.2%)	19 (56.9%)
Junior doctor	3 (4.1%)	0	1 (4.3%)	5 (14.7%)

**Table 2:** Response rates and career stages of mentors

	Pre-questionnaire: September 2022 (*n* = 40)	Post-questionnaire: April 2023 (*n* = 38)
Response rate	40/101 (39.6%)	38/101 (37.6%)
Specialty registrar	28 (70.0%)	22 (57.9%)
Consultant	12 (30.0%)	16 (42.1%)

### Setting of the scheme

**Fig. 1:**
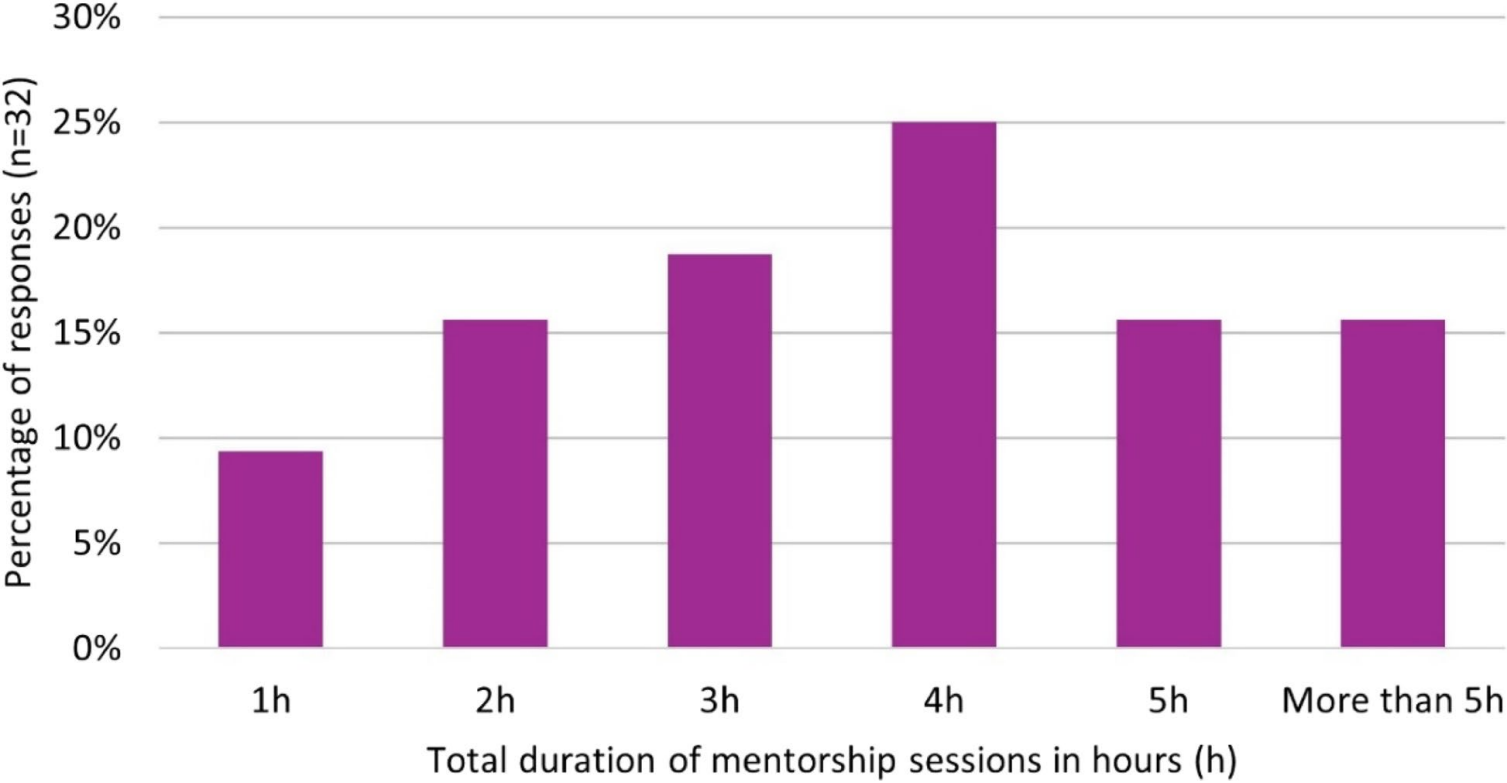
Cumulative duration of mentorship throughout the scheme as reported by mentees in the post-mentorship questionnaire

**Fig. 2:**
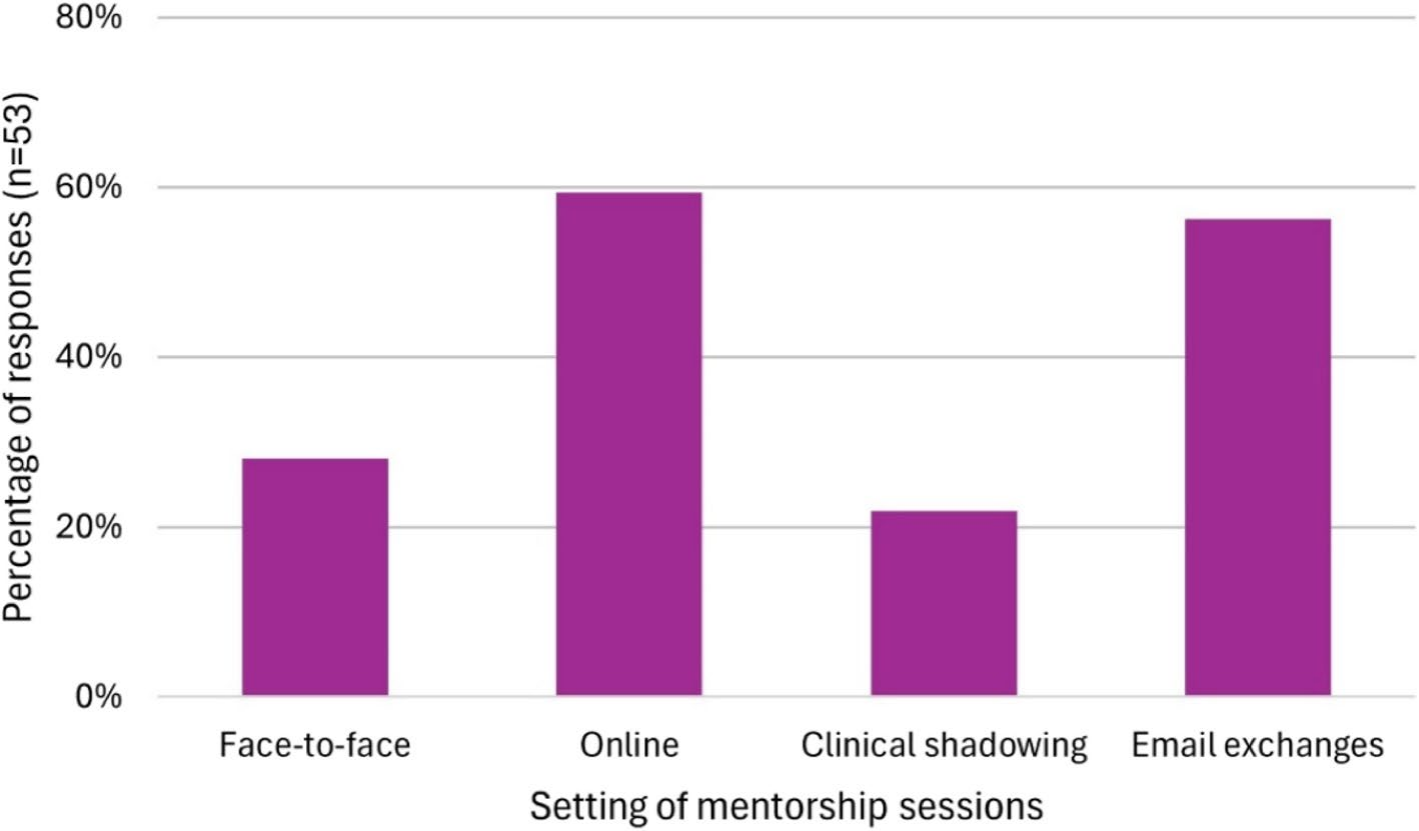
Most common setting for meetings throughout the scheme as reported by mentees in the post-mentorship questionnaire

Information on the length of the mentorship sessions and the meeting setting can be found in Fig. 1 and Fig. 2.

A very small number of participants reported having some of their sessions jointly with other mentees who were allocated to the same mentor.

### Knowledge of the scheme

Most mentees were interested in oncology and had specific aims and objectives for the BONUS Mentorship Scheme. Mentees’ clarity on what the mentorship scheme would look like prior to starting was divided (Table 3). There were no statistically significant differences in these parameters between pre-clinical students, clinical students and junior doctors (Additional file 1.7). Most mentors agreed or strongly agreed (*n* = 36, 75%) that they understood their mentees’ goals and expectations for the mentorship scheme.

**Table 3:** Pre-mentorship knowledge of the scheme as reported by mentees (*n* = 74)

	Agree or strongly agree	Neutral	Disagree or strongly disagree
“I am interested in oncology as a career”	81.1% (*n* = 60)	16.2% (*n* = 12)	2.7% (*n* = 2)
“I have a clear idea on what the mentorship scheme will look like”	29.7% (*n* = 22)	39.2% (*n* = 29)	31.1% (*n* = 23)
“I have specific aims and objectives for the mentorship scheme”	59.5% (*n* = 44)	36.1% (*n* = 26)	4.1% (*n* = 3)

In the pre-mentorship questionnaires, 78.4% of the mentees (*n* = 58) and 82.5% of the mentors (*n* = 47) agreed or strongly agreed that they would benefit from a written guide about undergraduate mentorship in oncology before starting the scheme. In the post-mentorship questionnaires, 62.5% of the mentees (*n *= 20) and 45.8% of the mentors (*n* = 22) agreed or strongly agreed with the same statement. Pre-mentorship concerns regarding the scheme were expressed in free text responses, e.g. *“Having never had a mentor before, I am unsure that my mentor knows the aims of the scheme and that we may potentially become slightly unsure of the direction our mentor–mentee relationship should take.”*

### Expectations and motivations

**Fig. 3:**
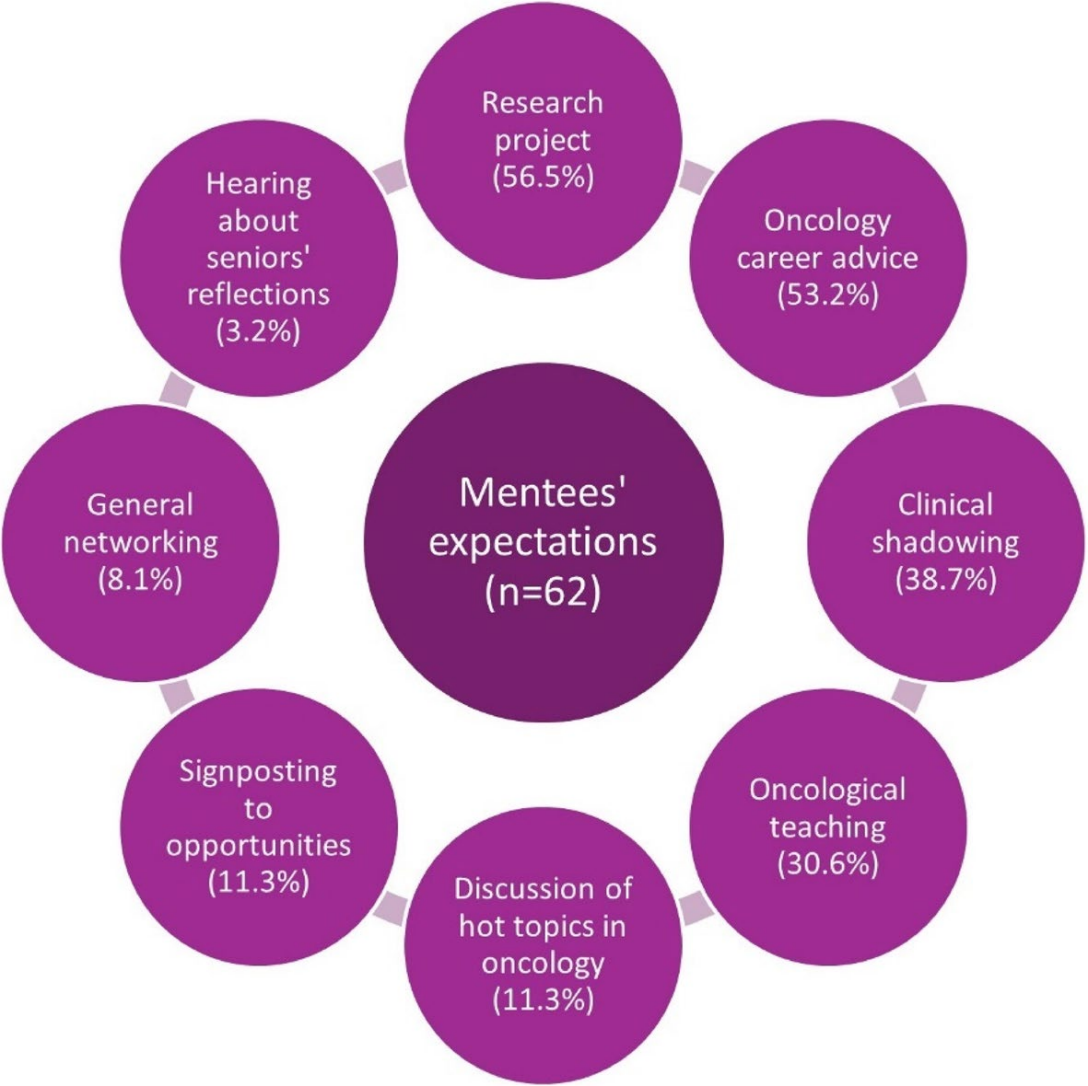
Expectations for the mentorship scheme as reported by mentees

A summary of mentees’ expectations can be found in Fig. 3. Most mentees expected to be involved in a research project, including audit and quality improvement. Desired oncology career advice ranged from explaining oncology career pathways to discussing specific specialty portfolio requirements. A typical response, as outlined by one participant was to “*discuss with my mentor about their career and sharing ideas for how I can develop and get involved in things at medical school to help further my own career in oncology.”* Another participant wished to *“shadow the mentor in their daily work in a hospital setting while working with cancer cases and hear anecdotes about mentor's experience of getting to where they are today.”*

When mentees were asked what they were most looking forward to, networking was the most frequent theme (*n* = 24, 38.1%). Mentees were also particularly excited to gain insight into the specialty (*n* = 17, 27.0%), gain research experience and outputs (*n* = 15, 23.8%), gain clinical experience (*n* = 11, 17.4%) and receive tailored career coaching (*n* = 10, 15.9%). Mentees generally referred to their lack of oncology exposure and therefore valued the opportunity for early experience. Mentees typically looked forward to *“acquiring hands-on experience in oncology (whether clinical or research) and getting advice on how to leverage potential opportunities to pursue interest in oncology even at pre-clinical stage of medicine.”*

**Fig. 4:**
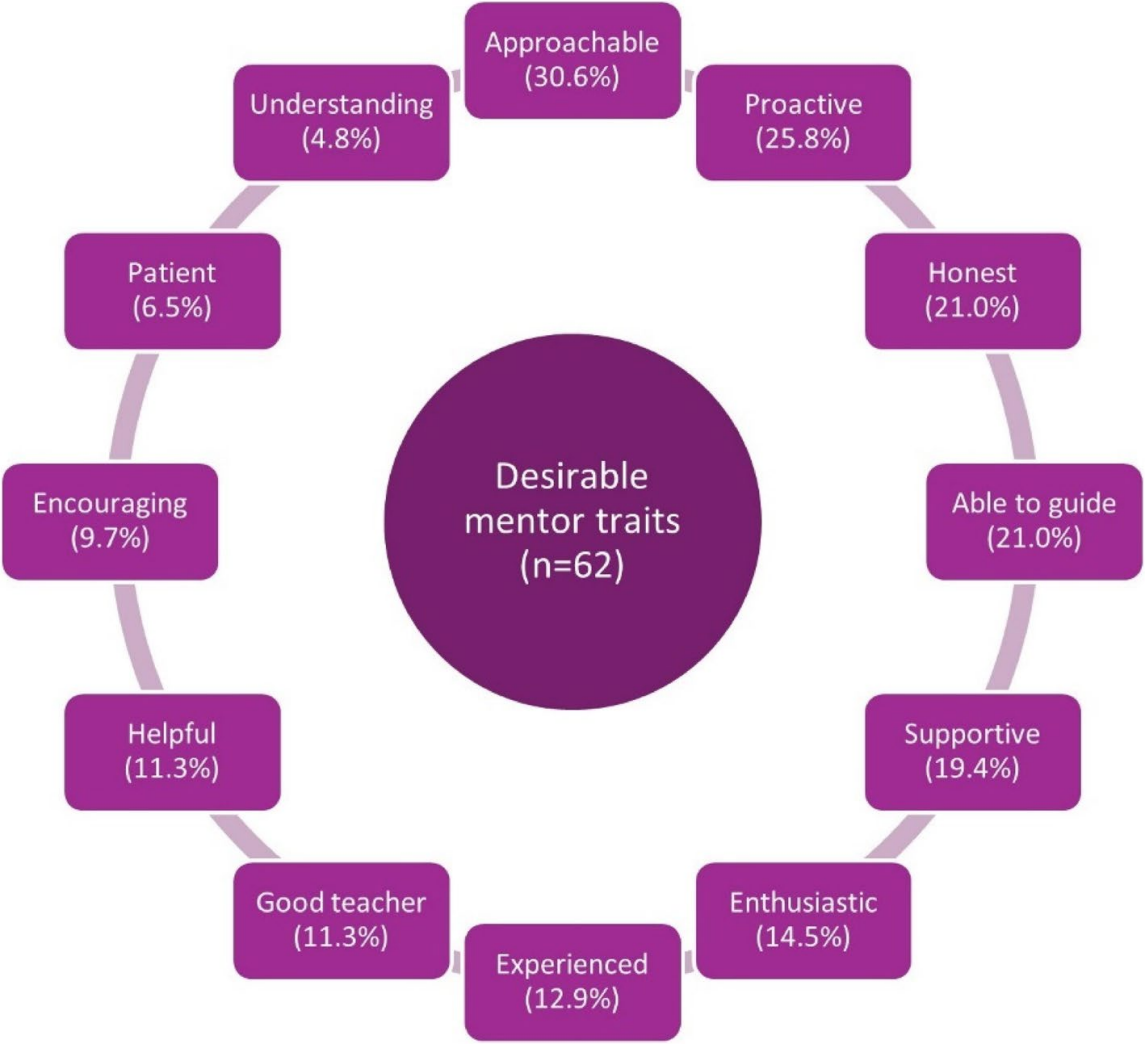
Desirable traits of a mentor as reported by mentees in the pre-mentorship questionnaire

The desirable attributes of a mentor reported by mentees are summarized in Fig. 4.

The mentors’ main motivation to become mentors was to promote oncology or inspire students and early career doctors into oncology (*n* = 20, 51.3%). Other common motivations included general junior support (*n* = 13, 33.3%) and personal satisfaction (*n* = 10, 25.6%). Several mentors undertook mentoring, primarily because of their interest in medical education and to develop their teaching, mentoring and leadership skills (*n* = 9, 23.1%). Some mentors were motivated to improve the experience of junior colleagues due to the lack of mentorship at an early stage of their own careers (*n* = 8, 20.5%). Conversely, some mentors’ motivation to mentor was to “give back”, as they had a positive experience as mentees in the past (*n* = 6, 15.4%). One mentor reported *“I have had great mentors in the past and feel that it is my time to repay it back.”* Finally, a small number of mentors simply wished to form relationships and expand their network (*n* = 3, 7.8%).

When the mentors were asked about specific ideas for mentorship, general career advice (*n* = 16, 45.7%) and the desire for it to be mentee-led (*n* = 14, 40.0%) were the most common themes. Mentors also suggested teaching (*n* = 11, 31.4%), clinical shadowing (*n* = 7, 20.0%), research project engagement (*n* = 7, 20.0%) and day-to-day oncology insight (*n* = 6, 17.1%).

Mentors planned to develop a good mentoring relationship from the start by agreeing with expectations and plans upfront (*n* = 19, 52.8%), being approachable (*n* = 11, 30.6%), developing rapport (*n* = 11, 30.6%), being open (*n* = 10, 27.8%) and being available (*n* = 8, 22.2%). Some mentors also believed that an initial face-to-face meeting (*n* = 6, 16.6%) and clear introductions (*n* = 3, 8.3%) were appropriate to kickstart a good mentoring relationship. Ideas for developing rapport included inviting the mentee for a coffee and providing a reachable contact number.

**Fig. 5:**
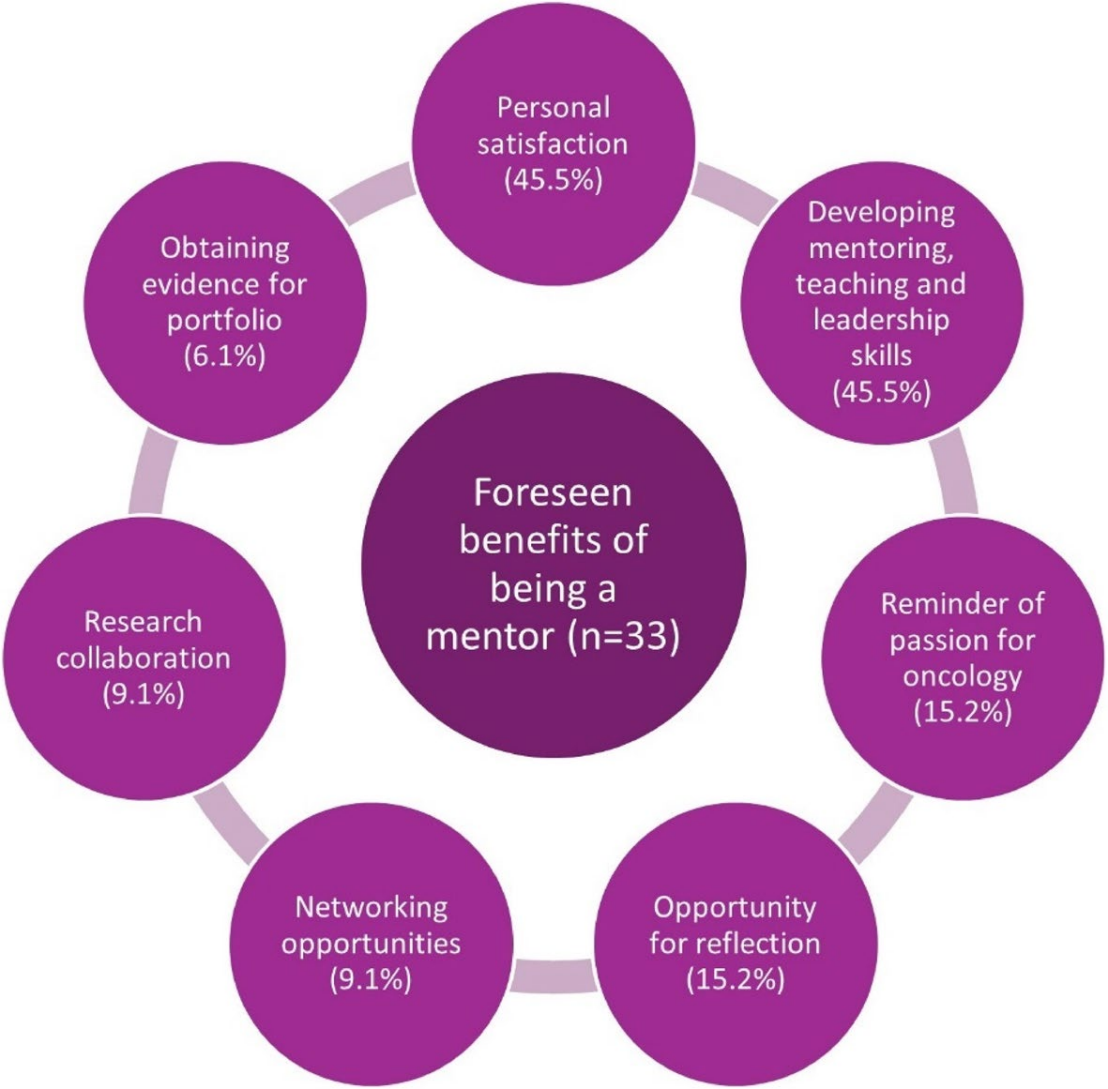
Benefits of being a mentor as reported by mentors in the pre-mentorship questionnaire

The foreseen benefits of being a mentor, as reported by mentors in the pre-mentorship questionnaires, are summarized in Fig. 5.

### Contents of the mentorship sessions

**Fig. 6:**
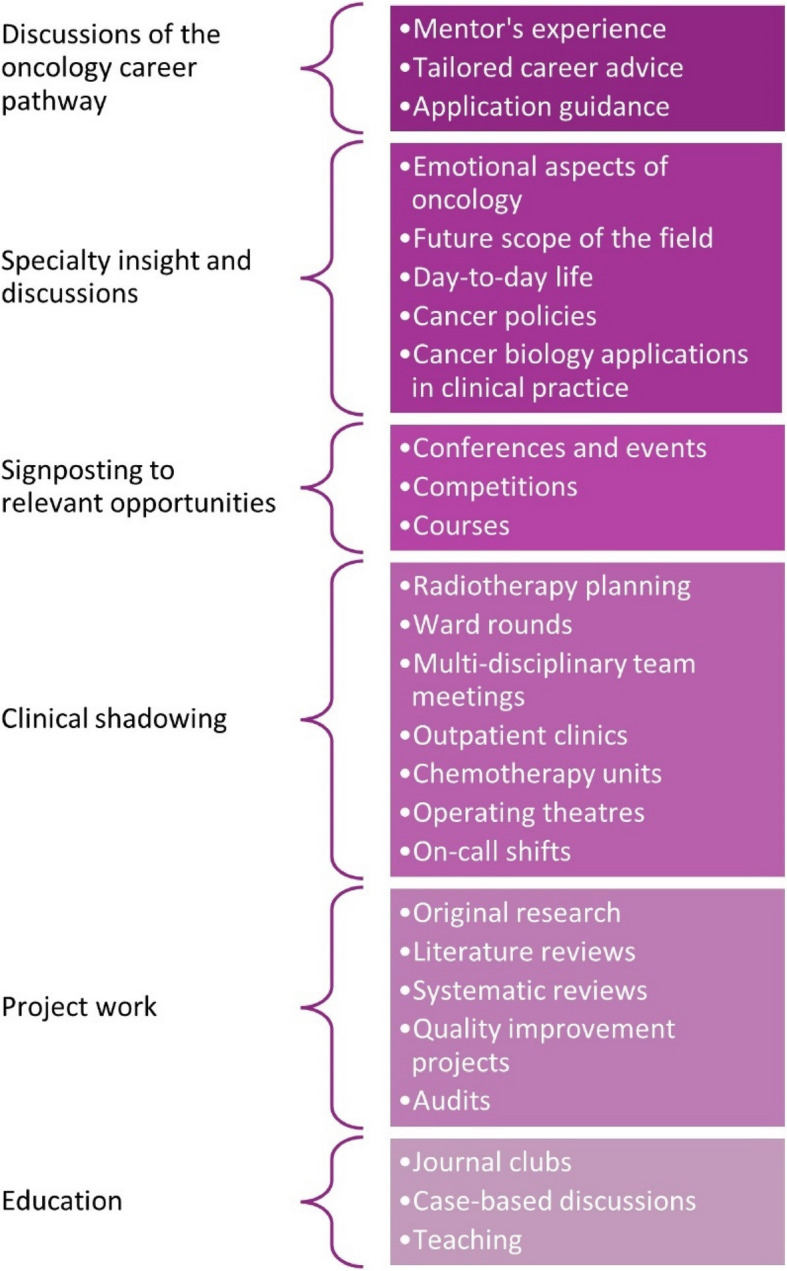
Ideas for undergraduate mentoring in oncology as inspired by the contents of the BONUS Mentorship Scheme

A summary of the contents of the BONUS Mentorship Scheme 2022–2023 is summarized in Fig. 6.

The most common reported content of the mentorship sessions involved discussions about the oncology career pathway (*n* = 12, 50.0%; *n* = 12, 60.0%; and *n* = 17, 56.7%, as reported in the first mid-mentorship, second mid-mentorship and the post-mentorship questionnaires, respectively). These included discussions of the mentor’s own professional pathway to becoming an oncologist and the lessons learned, as well as tailored career advice about the oncology training pathway for the mentees. Mentors generally offered to guide mentees in their future applications for specialty training and provided advice on intercalated degrees and study tips.

Other popular contents of the mentorship included signposting to current opportunities for mentees (*n* = 5, 20.8%; *n* = 2, 10.0%; and *n* = 7, 23.3%) and clinical shadowing (*n* = 5, 20.8%; *n* = 9, 45.0%; and *n* = 9, 30.0%). Mentees gained advice on opportunities for their training stage, such as attending relevant conferences or entering oncology-related prize competitions.

A few mentees spent their time engaging in projects (*n* = 4, 16.7%; *n* = 8, 40.0%; and *n* = 9, 30.0%), including research projects, literature reviews, systematic reviews, quality improvement projects and audits. A small number of mentees reported their work being published in peer-reviewed journals or being accepted for presentations at conferences and scientific meetings.

Some sessions involved discussions of oncology from a clinical perspective (*n* = 4, 16.7%; *n* = 7, 35.0%; and *n* = 8, 26.7%), during which medical students learned about the application of cancer biology in clinical practice. This approach was reported to be particularly valuable to pre-clinical students, who lacked clinical experience or knowledge.

Other mentorship sessions consisted of journal clubs (*n* = 2, 8.3%; *n* = 2, 10.0%; and *n* = 5, 16.6%), case discussions (*n* = 2, 8.3%; *n* = 3, 15.0%; and *n* = 5, 16.6%) and oncological teaching (*n* = 3, 12.5%; *n* = 6, 30.0%; and *n* = 4, 13.3%).

Teaching covered various topics, including cancer screening, oncological presentations, overviews of the cancer multi-disciplinary team meetings, oncology scoring systems, indications for chemotherapy and radiotherapy, immunotherapy, nutrition in oncology, overviews of cancer services in the National Health Service, oncological emergencies, acute oncology and evidence-based medicine.

Individualized sessions that mentors offered included summer research placement advice, doctoral degree application advice, help with an essay prize submission, planning a talk for a university society, attending a career evening and general pastoral support.

The mentor’s responses to the question about the specific content of their mentorship sessions in the post-mentorship questionnaires did not differ significantly from the answers provided by the mentees. For mentors, the most common content of the sessions was providing career advice (*n* = 21, 55.3%), followed by providing an opportunity for research (*n* = 20, 52.6%).

Nearly half of the responses described how the mentorship sessions provided the mentees with insight into oncology as a specialty (*n* = 17, 44.7%). This was done in a variety of ways and included discussions on the emotional impact of oncology, analysis of the future scope of the field, insights into day-to-day activities and a model weekly timetable, details of the medical and clinical oncology training programmes, and discussions of current cancer policies.

Several mentors provided clinical exposure for their mentees (*n* = 7, 18.4%), which included observing radiotherapy planning, attending clinics, and shadowing ward rounds and on-call shifts. Multiple mentors taught concepts to mentees during their sessions (*n* = 6, 15.8%), including introductions to radiotherapy, modes of action of chemotherapy and targeted therapy, as well as basic treatments for renal and colorectal cancers.

Other individualized sessions reported by mentors consisted of specific case discussions, critical appraisals of research papers and discussions of current issues in oncology.

### Feedback from participants

Mentee-reported outcomes after participation in the BONUS Mentorship Scheme are presented in Table 4. There were no statistically significant differences in these parameters between pre-clinical students, clinical students and junior doctors (Additional file 1.8).

**Table 4:** Post-mentorship feedback as reported by mentees

	Agree or strongly agree	Neutral	Disagree or strongly disagree
“I enjoyed the mentorship scheme” (*n* = 34)	91.2% (*n* = 31)	8.8% (*n* = 3)	0% (*n* = 0)
“I am more interested in a career in oncology as a result of the scheme” (*n* = 33)	78.8% (*n* = 26)	21.2% (*n* = 7)	0% (*n* = 0)
“Participating in the scheme was a valuable experience” (*n* = 34)	91.2% (*n* = 31)	8.8% (*n* = 3)	0% (*n* = 0)

Mentees and mentors reported online meetings to be the most useful (*n* = 19, 59.4% of mentees and *n* = 21, 43.8% of mentors), followed by face-to-face meetings (*n* = 6, 18.8% and *n* = 13, 27.1%) and clinical shadowing (*n* = 5, 15.6% and *n* = 6, 12.5%). There were no statistically significant differences in these parameters between mentees and mentors at different stages of their careers (Additional file 1.9). Email exchanges were reported to be the least useful by both groups (*n* = 10, 71.4% of mentees and *n* = 19, 39.6% of mentors).

Mentees generally strongly agreed or agreed (*n* = 16, 50.0%), 37.5% (*n* = 12) were neutral and 12.5% (*n* = 4) disagreed that they would have benefited from pre-arranged meetings with fellow mentees to share ideas. In terms of mentors, 35.3% (*n* = 18) strongly agreed or agreed, 47.1% (*n* = 24) were neutral, and 17.6% (*n* = 9) disagreed or strongly disagreed that they would have benefitted from prearranged meetings with fellow mentors to share ideas. There were no statistically significant differences in these parameters between mentees and mentors at different stages of their careers (Additional file 1.10).

Most mentees would have preferred to be part of the mentorship scheme during the clinical years of medical school (*n* = 23, 74.2%). Mentees generally plan to stay in touch with their mentor beyond the official timeframe of the scheme (*n* = 27, 84.4%), as so do mentors (*n* = 38, 79.2%). A total of 95.7% (*n* = 45) of the mentors would recommend their colleagues to become involved as mentors in the scheme and 95.8% (*n* = 46) would join the scheme again. There were no statistically significant differences in these parameters between mentees and mentors at different stages of their careers (Additional file 1.11).

In the post-mentorship questionnaire, mentees most commonly referred to overall exposure to oncology as a benefit of the scheme (*n* = 10, 40.0%). Other benefits included confirmation of oncology as the right future career choice (*n* = 7, 28.0%), having a future career plan and resources (*n* = 5, 20.0%) and gaining invaluable research experience (*n* = 5, 20.0%). Mentees described benefitting from new knowledge, new or additional clinical experience, detailed career discussions and networking opportunities. As an example, one mentee described the benefits as *“having more of a concrete idea about clinical oncology and plans to go down this route as it has all the elements I value in a career (research, clinical time, work/life balance)”*.

Mentors stated that research experience (*n* = 12, 34.3%) was one of the key benefits of the scheme for the mentees. Career building (*n* = 9, 25.7%), discussions of what a career in oncology is like (*n* = 9, 25.7%) and gaining specific specialist knowledge of oncology topics were also perceived benefits for the mentees. A few mentors believed that specialist clinical experience (*n* = 4, 11.4%) and networking (*n* = 3, 8.8%) were the main benefits for mentees. For example, one mentor described that their *“Mentee near end of medical school was able to attend an oncology careers evening I helped organise and signposted her to. I also gave networking and research opportunities with local oncologists.”*

Most mentors reported that personal satisfaction (*n* = 16, 48.5%) was the main benefit for them. Mentors also believed that the scheme promoted oncology as an attractive career choice for students and doctors (*n* = 6, 18.2%). For some mentors, reflection on their career as oncologists (*n* = 4, 12.1%) and the opportunity to develop their mentoring (*n* = 3, 9.1%) and teaching (*n* = 2, 6.1%) skills were the key acquired benefits. One mentor described: *“The inquisitive questions asked by the mentees always make me reflect on why I chose oncology and in a busy and pressurised time, help me to remember all the things I love about it!”.*

There were no specific areas of the mentorship scheme with which the mentees were dissatisfied. Individual responses for the areas of improvement included having better communication from the mentor, having more research and clinical shadowing opportunities, meeting face-to-face as opposed to online, having meetings more frequently and increasing the duration of the scheme. Many mentors referred to their own time constraints as the main obstacle of the mentorship scheme (*n* = 18, 48.6%). Some mentors reported that a lack of sufficient time, logistical issues in setting up access to systems and a lack of experience from students were obstacles to conducting research.

## Discussion

The benefits of early career mentorship are well established across different specialties [8]. A study on the previous cycle of the BONUS Mentorship Scheme demonstrated the benefits of participation for mentees and mentors, however specific content areas discussed within sessions were not studied. It was suggested that qualitative methodologies should be used to investigate this topic in future work [13]. Furthermore, previous qualitative studies on undergraduate mentoring revealed that there is poor knowledge of participant expectations [19]. In the current study, the lack of clarity regarding the aims of the BONUS Mentorship Scheme was highlighted by mentees prior to starting, and the participants agreed that they would benefit from a written guide about undergraduate mentorship in oncology. To this effect, we provide ideas that mentees and mentors can utilize for future mentoring (Fig. 6). The insights presented in this study can be utilized to streamline the content and guide future mentorships. To our knowledge, this is the first study to rigorously describe the contents of undergraduate mentorship sessions in oncology in the UK.

The findings of our mentorship scheme are informed by the Association for Medical Education in Europe (AMEE) Guide No. 167 on mentorship in health professions [17]. Our scheme was designed to follow the traditional one-on-one dyadic format, in which one mentor and one mentee work together as a pair. We found that some participants reported being interested in meeting fellow mentees or mentors to discuss ideas. Although not formally studied, meeting others could lead to group or peer mentoring, which have their own benefits [20]. In fact, individual survey responses referred to hosting joint mentorship sessions, for example with one mentor providing a teaching session to multiple mentees. For future cycles of the BONUS Mentorship Scheme, we plan to utilize online forum platforms, which participants can join voluntarily to discuss ideas and engage in group mentoring. Supported by the AMEE Guide No. 167, the online forums would have a potential to build “communities of practice”, in which a group of mentees and mentors would have a common interest in a topic, and come together to discuss and share ideas [21, 22].

The reported obstacles of our scheme align well with the challenges defined in the AMEE Guide No. 167, especially with respect to commitment, time constraints and competing priorities. The issue of time constraints may also reflect poor response rates to questionnaires.

Online meetings were reported to be the most useful setting for the sessions, which aligns with recent studies on virtual mentoring [23, 24]. The virtual setting may be better suited to fit personal commitments and time constraints. Online mentoring may also be more suitable for mentor–mentee pairs across different geographical locations and those with other accessibility requirements. It is possible that some participants graded online sessions as most useful due to lack of experience with other mentoring settings in our scheme. Future work could evaluate the benefits of virtual mentoring in early career oncology in comparison to face-to-face meetings.

The emerging theme of the mentorship sessions was the beneficial opportunity to discuss career pathways. This was evident in the mentees’ expectations for the scheme. Previous studies have reported a general lack of information on a career in oncology during medical training [12, 25]. Mentees reported being more interested in a career in oncology after participating in the scheme. Although the opportunity for career guidance may also be available via other options, such as student-selected placements, taster weeks or career fairs, the one-to-one nature of mentoring provided added value for personalization. As informed by the AMEE Guide No. 167, the personalized aspects of mentoring have the benefit of the “zone of proximal development” concept, in which the more experienced mentor provides tailored guidance to the mentee to help them advance in their career [17].

Another key aspect of the mentorship sessions was enabling research projects. The desire to become involved in research at an early stage is common among medical students and early career doctors [26]. Mentorship plays an influential role in medical students’ overall experience with research [27]. Challenges in accessing knowledge and opportunities in research and academic medicine are well recognized [28, 29]. A lack of meaningful mentorship is a barrier to early career research [30]. The BONUS Mentorship Scheme successfully enabled oncology-specific research activity among a minority of participants.

Despite positive research outputs, we found a mismatch in mentee and mentor expectations for research projects. Fifty-six percent of mentees had an expectation of becoming involved in research. Only 20% of mentors pre-emptively suggested research projects as a potential idea for the mentorship. The proportion of mentees who ultimately reported being involved in projects varied from 16.6% to 40% across mid- and post-mentorship questionnaires. The individually reported barriers to research included time constraints, logistical issues, and the inexperience of the early year mentees. It may be that mentors can signpost to and provide career advice on academic pathways, but offering projects may be an expectation beyond the remits of the scheme. In the subsequent cycles of the BONUS Mentorship Scheme, we will seek to ask mentees to state their preference for research and match them with research-minded mentors. This may bridge the gap between mentees’ demand for research and mentors’ ability to supply projects.

Our study is at risk of nonresponse bias due to low response rates in the mid-mentorship questionnaires. Those who had a positive experience may have been more likely to respond to questionnaires. There is a selection bias within the cohort that applied to the scheme, as the scheme was primarily advertised by BONUS, a society whose target audience may already express high interest in oncology. This was evident in our pre-mentorship questionnaire. Web-based survey methods with open-ended questions provide only basic qualitative insights and may have reduced the richness of the data. The questionnaire used was unvalidated and was developed specifically for use in this scheme.

Future work could utilize validated instruments, reflective assignments, interviews and focus groups to obtain in-depth insights into the outcomes of the BONUS Mentorship Scheme [31]. Long-term follow-up of mentees would be of interest to assess if any had opted for specialty training in oncology and whether any of the research experience translated into scholarship in terms of presentations and publications. As more undergraduate mentorship schemes become available in other specialties, it would be of interest to compare them and identify pertinent points which differentiate the BONUS Mentorship Scheme with respect to its focus in oncology.

## Conclusions

In this study, we highlighted the contents of the BONUS Mentorship Scheme as ideas for mentoring in oncology. The proposed ideas span across the primary domains of career pathway discussions, specialty insight, signposting to opportunities, clinical shadowing, project work and education.

We identified several benefits of the programme and the reasons for high satisfaction levels among participants. For mentees, these include early exposure to oncology, confirmation of oncology as the right career choice, and research experience. For mentors, the reported benefits are personal satisfaction, the promotion of oncology and opportunities for reflection.

We described that one of the main expectations for mentees is to become involved in research; however, there is a mismatch in research-related expectations between mentors and mentees. As such, future work is needed in this area to effectively facilitate oncology research among medical students and early career doctors.

We found that online mentorship meetings were perceived to be most suitable for the majority of mentees. This presents a good opportunity for establishing national, and even international mentorships in the field.

Our study contributes to implementing a comprehensive guide for ideas for mentoring in oncology, with the aim of streamlining the mentorship process, and ensuring consistency and clarity in objectives. Our findings confirm the success of the BONUS Mentorship Scheme, which continues to be an established annual initiative. Future work could follow-up mentees until they become specialty trainees to establish whether participants have chosen a career in oncology.

## Supplementary Information

Supplementary Material 1.

## Data Availability

The datasets analyzed during the current study are available from the corresponding author upon reasonable request.

## Abbreviations

BONUS: British Oncology Network for Undergraduate Societies
UK: United Kingdom
AMEE: Association for Medical Education in Europe
