# Supplementary material for: Ideas for mentorship in oncology for medical students and early career doctors: insights from a UK-wide oncology mentorship scheme
Source: BMC Med Educ. 2025 Jul 21;25:1091. doi: 10.1186/s12909-025-07219-2 (PMC12281856; doi:10.1186/s12909-025-07219-2)
Supplement: Supplementary file 1 — Supplementary Material 1. [file 12909_2025_7219_MOESM1_ESM.docx]

**Additional file 1**

**1.1 Pre-Mentorship Questionnaire for Mentees**

Please complete this questionnaire, so the BONUS committee can receive feedback and assess the outcomes of the National Oncology Mentorship Scheme. All the questions are optional and anonymous.

If you have any questions, please contact BONUS at [bonus.oncology@gmail](mailto:bonus.oncology@gmail).com

* Indicates required question

1. What is your career stage? Select only one.

Pre-clinical medical student

Clinical medical student

Junior doctor

2. I am interested in oncology as a career. Select only one.

Strongly disagree

Disagree

Neutral

Agree

Strongly agree

3. Where have you gained experience in oncology so far? Check all that apply.

Lectures and formal teaching

Oncology placement at medical school

Other clinical placement at medical school

Elective/Student Selected Placement

Research project

Intercalation/Masters/PhD

Volunteering

Clinical job as a junior doctor

Other: (free text)

4. I have a clear idea on what the mentorship scheme will look like. Select only one.

Strongly disagree

Disagree

Neutral

Agree

Strongly agree

5. What are you expecting your mentorship sessions to look like? (Free text)

Please describe in detail any ideas you have for your sessions.

6. I have specific aims and objectives for the mentorship. Select only one.

Strongly disagree

Disagree

Neutral

Agree

Strongly agree

7. What are your aims and objectives for the mentorship scheme? (Free text)

8. What are you most looking forward to in the mentorship scheme? (Free text)

9. I am concerned about some aspects of the mentorship scheme. Select only one.

Strongly disagree

Disagree

Neutral

Agree

Strongly agree

10. What are your concerns at this stage, if you have any, about the mentorship scheme? (Free text)

11. What are you looking for in a mentor? (Free text)

12. I would benefit from a written guide about undergraduate mentorship in oncology before starting. Select only one.

Strongly disagree

Disagree

Neutral

Agree

Strongly agree

13. Any other comments before you begin your mentorship. (Free text)

14. I consent for my answers to this anonymous questionnaire to be used in research presentations and publications* Select only one.

Yes

No

**1.2 Pre-Mentorship Questionnaire for Mentors**

Please complete this questionnaire, so the BONUS committee can receive feedback and assess the outcomes of the National Oncology Mentorship Scheme. All the questions are optional and anonymous.

If you have any questions, please contact BONUS at bonus.oncology@gmail.com

* Indicates required question

1. What is your career stage? Select only one.

Consultant or equivalent

Specialty Registrar or equivalent

2. Have you previously participated or are you currently participating in another formal mentorship scheme? Select only one.

Yes

No

3. Were you a mentor in the previous cycle of the BONUS National Oncology Mentorship Scheme (in 2021/22)? Select only one.

Yes

No

4. What is your motivation for becoming a mentor? (Free text)

5. Which of these areas do you feel you may be able to bring or incorporate into your mentoring sessions? Check all that apply.

Clinical experience or shadowing

Careers advice

Discussion of case studies

Discussion of research papers

Support with research opportunities

Teaching in oncology

Networking

Other: (free text)

6. What are your specific ideas for mentorship sessions? (Free text)

7. How do you think your mentee will benefit from the mentorship? (Free text)

8. What obstacles do you think you might encounter during the mentorship? (Free text)

9. How will you develop a good mentoring relationship from the start? (Free text)

10. What personal benefits might you get as a result of being a mentor? (Free text)

11. I would benefit from a written guide about undergraduate mentorship in oncology before starting. Select only one.

Strongly disagree

Disagree

Neutral

Agree

Strongly agree

12. Any other comments before you begin your mentorship (Free text)

13. I consent for my answers to this anonymous questionnaire to be used in research presentations and publications* Select only one.

Yes

No

**1.3 Mid-Mentorship Questionnaire**

Please complete this questionnaire, so the BONUS committee can receive feedback and assess the outcomes of the National Oncology Mentorship Scheme. All the questions are optional and anonymous.

If you have any questions, please contact BONUS at bonus.oncology@gmail.com

* Indicates required question

1. What is your career stage? Select only one.

Pre-clinical medical student

Clinical medical student

Junior doctor

2. How many hours of mentoring have you had so far? Select only one.

0

Less than 1

1

2

3

Other: (free text)

3. What was the setting of your mentorship so far? Please select multiple options if applicable. Check all that apply.

Online meeting

Face-to-face meeting

Shadowing in clinical setting

Email exchange

Other: (free text)

4. I am enjoying the mentorship scheme. Select only one.

Strongly agree

Agree

Neutral

Disagree

Strongly disagree

5. Which areas have you covered in your mentorship so far? Please select multiple options if applicable. Check all that apply.

Insights into a career in oncology

Experience of oncology in clinical setting

Discussion of case studies and research papers

General CV/careers advice

Engagement in research projects

Teaching on oncology topics

Networking

Other: (free text)

6. What specifically have you done in your mentorship sessions so far? Please describe the contents of your mentorship to date. (Free text)

7. What benefits have you gained from the mentorship so far? (Free text)

8. What are you looking forward to for the rest of the mentorship? (Free text)

9. What do you wish to be different for the rest of the mentorship? (Free text)

10. Any other comments? (Free text)

11. I consent for my answers to this anonymous questionnaire to be used in research presentations and publications* Select only one.

Yes

No

**1.4 Post-Mentorship Questionnaire for Mentees**

Please complete this questionnaire, so the BONUS committee can receive feedback and assess the outcomes of the National Oncology Mentorship Scheme. All the questions are optional and anonymous.

If you have any questions, please contact BONUS at bonus.oncology@gmail.com

* Indicates required question

1. What is your career stage? Select only one.

Pre-clinical medical student

Clinical medical student

Junior doctor

2. How many hours of mentoring have you had? (Free text)

3. What was the setting of your mentorship? Please select multiple options if applicable. Check all that apply.

Online meetings

Face-to-face meetings

Shadowing in clinical setting

Email exchanges

Other: (free text)

4. Which setting did you feel was the most useful? Select only one.

Online meetings

Face-to-face meetings

Shadowing in clinical setting

Email exchanges

Other: (free text)

5. Which setting did you feel was the least useful? Select only one.

Online meetings

Face-to-face meetings

Shadowing in clinical setting

Email exchanges

Other: (free text)

6. I enjoyed the mentorship scheme. Select only one.

Strongly agree

Agree

Neutral

Disagree

Strongly disagree

7. I am more interested in a career in oncology as a result of the mentorship scheme. Select only one.

Strongly agree

Agree

Neutral

Disagree

Strongly disagree

8. The mentorship scheme was a valuable experience. Select only one.

Strongly agree

Agree

Neutral

Disagree

Strongly disagree

9. Which areas have you covered in your mentorship sessions? Please select multiple options if applicable. Check all that apply.

Insights into a career in oncology

Experience of oncology in clinical setting

Discussion of case studies and research papers

General CV/careers advice

Engagement in research projects

Teaching on oncology topics

Networking

Other: (free text)

10. What specifically have you done in your mentorship sessions? Please describe the contents of your mentorship. We are looking for descriptions of shadowing experience, career discussions, research papers you've worked on, etc. (Free text)

11. What have you learnt from your mentor? (Free text)

12. What benefits have you gained from the mentorship? (Free text)

13. What should have been different or improved? (Free text)

14. Would you have preferred to have been part of the mentorship scheme during pre-clinical or clinical years? Select only one.

Pre-clinical

Clinical

15. I plan to stay in touch with my mentor beyond the official timeframe of the scheme. Select only one.

Yes

No

Other:

16. I would have benefited from a written guide about undergraduate mentorship in oncology before starting. Select only one.

Strongly agree

Agree

Neutral

Disagree

Strongly disagree

17. I would have benefited from pre-arranged meetings with fellow mentees to share ideas. Select only one.

Strongly agree

Agree

Neutral

Disagree

Strongly disagree

18. I would be interested to join the mentorship scheme as a mentee again. Select only one.

Yes

No

19. I would recommend the mentorship scheme to a colleague. Select only one.

Yes

No

20. Any other comments? (Free text)

21. I consent for my answers to this anonymous questionnaire to be used in research presentations and publications* Select only one.

Yes

No

**1.5 Post-Mentorship Questionnaire for Mentors**

Please complete this questionnaire, so the BONUS committee can receive feedback and assess the outcomes of the National Oncology Mentorship Scheme. All the questions are optional and anonymous.

If you have any questions, please contact BONUS at bonus.oncology@gmail.com

* Indicates required question

1. What is your career stage? Select only one.

Consultant or equivalent

Specialty Registrar or equivalent

2. How many hours of mentoring did you provide to your mentees? (Free text)

3. What was the setting of your mentorship? Please select multiple options if applicable. Check all that apply.

Online meetings

Face-to-face meetings

Shadowing in clinical setting

Email exchanges

Other: (free text)

4. Which setting did you feel was the most useful? Select only one.

Online meetings

Face-to-face meetings

Shadowing in clinical setting

Email exchanges

Other: (free text)

5. Which setting did you feel was the least useful? Select only one.

Online meetings

Face-to-face meetings

Shadowing in clinical setting

Email exchanges

Other: (free text)

6. I understood my mentees' goals and expectations for the mentorship scheme. Select only one.

Strongly disagree

Disagree

Neutral

Agree

Strongly agree

7. Which of these areas have you incorporated into your mentoring sessions? Check all that apply.

Clinical experience or shadowing

Careers advice

Discussion of case studies

Discussion of research papers

Support with research opportunities

Teaching in oncology

Networking

Other: (free text)

8. What were your specific ideas for mentorship sessions and what was achieved? We are looking for descriptions of the specific contents of the mentoring you provided. (Free text)

9. How has your mentee benefited from the mentorship? (Free text)

10. What obstacles have you encountered during the mentorship? (Free text)

11. What personal benefits did you get as a result of being a mentor? (Free text)

12. I plan to stay in touch with my mentee(s) beyond the official timeframe of the scheme. Select only one.

Yes

No

Other:

13. I would have benefited from a written guide about undergraduate mentorship in oncology before starting. Select only one.

Strongly disagree

Disagree

Neutral

Agree

Strongly agree

14. I would have benefitted from prearranged meetings with fellow mentors to share ideas. Select only one.

Strongly disagree

Disagree

Neutral

Agree

Strongly agree

15. I would recommend my colleagues to get involved as mentors in this mentorship scheme. Select only one.

Yes

No

16. I would join the mentorship scheme as a mentor again. Select only one.

Yes

No

17. Any other comments (Free text)

18. I consent for my answers to this anonymous questionnaire to be used in research presentations and publications* Select only one.

Yes

No

**1.6 Prior experience of oncology among pre-clinical, clinical and junior doctor mentees prior to starting of the scheme**

|  | Lectures and formal teaching | Prior involvement in a research project | Oncology placement at medical school | Other non-oncology placements at medical school | Volunteering | Participation in the previous cycle of the scheme |
| --- | --- | --- | --- | --- | --- | --- |
| Pre-clinical student (n=31) | 29 (93.5%) | 7  (22.6%) | 0 | 3  (9.7%) | 5  (16.1%) | 2  (6.5%) |
| Clinical student (n=40) | 37 (92.5%) | 13  (32.5%) | 14  (35.0%) | 16  (53.3%) | 6  (15.0%) | 9  (22.5%) |
| Junior doctor (n=3) | 2  (66.7%) | 1  (33.3%) | 1  (33.3%) | 0 | 0 | 0 |
| p-value* | 0.2883 | 0.6038 | 0.0001 | 0.0003 | 1 | 0.1721 |

*As determined by Fisher’s exact test

**1.7 Pre-mentorship knowledge of the scheme as reported by pre-clinical, clinical and junior doctor mentees**

|  | “I am interested in oncology as a career” | | | “I have a clear idea on what the mentorship scheme will look like” | | | “I have specific aims and objectives for the mentorship scheme” | | |
| --- | --- | --- | --- | --- | --- | --- | --- | --- | --- |
|  | Agree* | Neutral | Disagree† | Agree | Neutral | Disagree | Agree | Neutral | Disagree |
| Pre-clinical student | 27  (87.1%) | 4 (12.9%) | 0 | 10 (32.3%) | 11 (35.4%) | 10 (32.3%) | 19 (61.3%) | 11 (35.5%) | 1 (3.2%) |
| Clinical student | 31 (77.5%) | 8 (20.0%) | 1  (2.5%) | 11 (27.5%) | 16 (40.0%) | 13 (32.5%) | 24 (61.5%) | 13 (33.3%) | 2 (5.2%) |
| Junior doctor | 2 (66.6%) | 0 | 1  (33.3%) | 1 (33.3%) | 2 (66.6%) | 0 | 1 (33.3%) | 2 (66.6%) | 0 |
| p-value‡ | 0.1627 | | | 0.8716 | | | 0.8140 | | |

*****Includes “agree” and “strongly agree”
†Includes “disagree” and “strongly disagree”
‡As determined by Fisher’s exact test

**1.8 Post-mentorship feedback as reported by pre-clinical, clinical and junior doctor mentees**

|  | “I enjoyed the mentorship scheme” | | | “I am more interested in a career in oncology as a result of the scheme” | | | “Participating in the scheme was a valuable experience” | | | |
| --- | --- | --- | --- | --- | --- | --- | --- | --- | --- | --- |
|  | Agree* | Neutral | Disagree† | Agree | Neutral | Disagree | | Agree | Neutral | Disagree |
| Pre-clinical student | 9  (90.0%) | 1 (10.0%) | 0 | 8 (80.0%) | 2 (20.0%) | 0 | | 9  (90.0%) | 1 (10.0%) | 0 |
| Clinical student | 17 (89.5%) | 2 (10.5%) | 0 | 12 (75.0%) | 4 (25.0%) | 0 | | 17 (89.5%) | 2 (10.5%) | 0 |
| Junior doctor | 5 (100%) | 0 | 0 | 4 (80.0%) | 1 (20.0%) | 0 | | 5 (100%) | 0 | 0 |
| p-value‡ | 1 | | | 1 | | | 1 | | | |

*****Includes “agree” and “strongly agree”
†Includes “disagree” and “strongly disagree”
‡As determined by Fisher’s exact test

**1.9 The most useful mentoring settings as reported by pre-clinical, clinical and junior doctor mentees, and specialty registrar and consultant mentors**

|  | Online | Face-to-face | Clinical shadowing |
| --- | --- | --- | --- |
| Pre-clinical student (n=8) | 7 (87.5%) | 0 | 1 (12.5%) |
| Clinical student (n=19) | 9 (47.4%) | 5 (26.3%) | 4 (21.0%) |
| Junior doctor (n=5) | 3 (60.0%) | 0 | 0 |
| p-value* | 0.2113 | 0.2358 | 0.8075 |
| Specialty Registrar or equivalent (n=26) | 11 (42.3%) | 7 (26.9%) | 3 (11.5%) |
| Consultant or equivalent (n=23) | 12 (52.2%) | 5 (21.7%) | 3 (13.0%) |
| p-value† | 0.6863 | 0.9296 | 1 |

*As determined by Fisher’s exact test
†As determined by Chi-square test

**1.10 Post-mentorship responses to “I would have benefited from pre-arranged meetings with fellow mentees or mentors to share ideas”**

|  | Agree or strongly agree | Neutral | Disagree or strongly disagree |
| --- | --- | --- | --- |
| Pre-clinical student (n=9) | 6 (66.6%) | 3 (33.3%) | 0 |
| Clinical student (n=18) | 8 (44.4%) | 7 (38.9%) | 3 (16.7%) |
| Junior doctor (n=5) | 2 (40.0%) | 2 (40.0%) | 1 (20.0%) |
| p-value* | 0.7309 | | |
| Specialty Registrar or equivalent (n=28) | 12 (42.9%) | 10 (35.7%) | 6 (21.4%) |
| Consultant or equivalent (n=22) | 6 (27.3%) | 14 (63.6%) | 2 (9.1%) |
| p-value† | 0.1350 | | |

*As determined by Fisher’s exact test
†As determined by Chi-square test

**1.11 Post-mentorship feedback as reported by pre-clinical, clinical and junior doctor mentees, and Specialty Registrar and Consultant mentors**

|  | “I plan to stay in touch with my mentor/mentee beyond the official timeframe of the scheme” | “I would recommend the scheme to my colleagues” | “I would join the scheme again” |
| --- | --- | --- | --- |
| Pre-clinical student | 8 (80.0%) | 8 (88.9%) | 10 (90.1%) |
| Clinical student | 14 (87.5%) | 18 (100%) | 16 (94.1%) |
| Junior doctor | 5 (100%) | 5 (100%) | 4 (80.0%) |
| p-value* | 0.6313 | 0.4375 | 0.7258 |
| Specialty Registrar or equivalent | 23 (82.1%) | 27 (100%) | 27 (96.4%) |
| Consultant or equivalent | 17 (80.1%) | 20 (90.1%) | 20 (90.1%) |
| p-value† | 1 | 0.3822 | 0.8290 |

*As determined by Fisher’s exact test
†As determined by Chi-square test
